# Comparison of Changes in Gut Microbiota in Wild Boars and Domestic Pigs Using 16S rRNA Gene and Metagenomics Sequencing Technologies

**DOI:** 10.3390/ani12172270

**Published:** 2022-09-01

**Authors:** Limin Wei, Weiying Zhou, Zhaojing Zhu

**Affiliations:** 1 Chongqing Key Laboratory of High Active Traditional Chinese Drug Delivery System, Chongqing Medical and Pharmaceutical College, Chongqing 401331, China; 2 College of Pharmacy, Chongqing Medical University, Chongqing 400016, China; 3 Chongqing Key Laboratory of Drug Metabolism, Chongqing Medical University, Chongqing 400016, China

**Keywords:** wild boars, domestic pigs, gut microbiota, 16S rRNA V3-V4 sequencing, 16S rRNA full-length sequencing, metagenomics

## Abstract

**Simple Summary:**

The microbiota co-evolves with the host and plays an important role in the host's health, immunity, and nutrient absorption. Wild boars are the ancestors of domestic pigs. During the long evolutionary process, the physiological structure and living habits of modern pigs have undergone tremendous changes. However, there are few studies on the evolution of gut microbiota of wild boars and domestic pigs. In this study, by comparing the changes in the composition and function of the gut microbiota of wild boars and domestic pigs, it was found that there were significant differences between the two groups, which indicated that the gut microbiota had changed during the evolution process. This study provides some data references for the evolution of gut microbiota.

**Abstract:**

Gut microbiota diversity is a result of co-evolution between microorganisms and their hosts. However, there are few studies on the evolution of the gut microbiota of wild boars and domestic pigs. Therefore, this study aimed to analyze the composition and function of the gut microbiota of wild boars and domestic pigs using 16S rRNA gene V3-V4 region sequencing, 16S rRNA gene full-length sequencing, and metagenomic sequencing. This study showed that after a long evolution, as compared to wild boars, the domestic pigs exhibited significantly increased relative abundances of *Lactobacillus*, *Lactobacillus reuteri*, *Lactobacillus johnsonii*, *Lactobacillus sp.DJF_WC5*, and *Lactobacillus*; *s_uncultured bacterium*, while the relative abundances of *Bifidobacterium* and *Methanococcaceae* decreased significantly. In addition, the relative abundances of “carbohydrate metabolism”, “starch and sucrose metabolism”, “valine, leucine, and isoleucine biosynthesis”, “lysine biosynthesis”, and starch-degrading CAZymes were significantly increased in the domestic pigs, while the relative abundances of “environmental adaptation”, “immune system”, “fatty acid degradation and synthesis”, and cellulose-hemicellulose-degrading CAZymes were significantly increased in the wild boars. Finally, the diversity of ARGs and the “antimicrobial resistance genes” in domestic pigs also increased significantly. This study illustrates that the gut microbiota composition and function of wild boars and domestic pigs changed during the long evolution process. These findings provide a basic research theory for the evolution of gut microbiota and the treatment of health and disease.

## 1. Introduction

The long-term co-evolution of gut microbiota and its respective hosts has enabled the establishment of a mutually beneficial homeostatic system [1]. The gut microbiota forms the intestinal barrier against pathogenic bacteria and also plays an important role in nutrient metabolism, growth, immunity, and regulation of its host [2,3,4,5]. Previous studies have described that during long-term evolution, two-thirds of the dominant families of microorganisms found in the gut of humans and apes converged into a last common ancestor which lived approximately 15 million years ago [6,7]. The gut microbiota has been associated with human hosts for a long period, with less variation happening within the families *Bacteroidaceae* and *Bifidobacteriaceae* [6,7]. During evolution, the genomes of the cell nucleus and the mitochondrion as well as the gut microbiome of hominids diverged synchronously [8,9]. However, due to the lack of a representative of an existing ancestral microbiota, the origin and evolution of host-specific gut microorganisms has not been fully elucidated, which has hindered the progress of gut microbiota research.

Wild boars were among the first wild animals to be domesticated by early humans. During the long domestication process of wild boars, a series of structural and physiological changes occurred [10]. For instance, compared with ancient wild boars, modern pigs have poor disease resistance, longer intestines, and higher growth rate [11,12]. Interestingly, pigs and humans have several similarities regarding their gastrointestinal anatomical structure, physiological characteristics, and gut microbiota composition [13,14,15]. Therefore, pigs and wild boars constitute an ideal mammalian model to study the origin and evolution of the gut microbiota.

However, only a few studies have attempted to explore the differences in the gut microbiota of wild boars and domestic pigs. Thus, the present study aimed to analyze and compare the gut microbiota composition of wild boars and domestic pigs using the 16S rRNA gene V3-V4 region sequencing, full-length 16S rRNA gene sequencing, and metagenomics techniques, intending to differentiate gut microbiota and KEGG metabolic function in the process of evolution.

## 2. Materials and Methods

### 2.1. Study Design, Sampling

Wild boars’ (Changbai Mountain wild boars) feces (*n* = 8) and domestic pigs’ (Duroc) feces (*n* = 7) were collected from Sichuan Jianyang pig farms. Approximately 30 g of feces was taken from the animal’s anus into a 40 mL sterile feces collection box and then quickly transferred into a liquid nitrogen container. Finally, it was placed in a −80 °C freezer for long-term storage.

The wild boars in this experimental farm were introduced from the northeast wild boar experimental farm, and their original parents were wild boar cubs captured on the Changbai mountain in northeast China in 2010. The wild boars used in this experiment were purebred of the 6th generation of the northeast wild boar farm and were raised in this experimental pig farm for 3.5–4 years. The wild boars and domestic pigs collected in this experiment were divided into two identical pens, each with 8 and 7 pigs, respectively (their sex and age were shown in the Table S2-sheet1), and the breeding environment and management of the pens were consistent. Their diets were similar, consisting of corn and soybean meal-based formulated feeds (Table S1) three times a day, 2 kg each time. All animals in this experiment were healthy without the use of any antibiotics.

Relative to 16S rRNA full-length sequencing, the 16S rRNA V3-V4 sequencing fragment is short, has low base mismatch rate, and higher accuracy [16,17,18], although only genus level could be identified [19]. In comparison, 16S rRNA full-length sequencing can compensate for the short sequencing depth of 16S rRNA V3-V4, which can classify more of the species level and higher species-level accuracy [20,21]. Therefore, combining the advantages and disadvantages of the two sequencing technologies, 16S rRNA V3-V4 sequencing was selected in this study for phylum, family, and genus level analysis of the gut microbiota, and 16S rRNA full-length sequencing was used for species-level analysis. Metagenomic technology was used for functional analysis of the gut microbiota.

### 2.2. DNA Extraction, 16SrRNA V3-V4 and Full-Length Sequencing

The DNA of microorganisms in fecal samples was extracted using the TIANamp Stool DNA Kit (TIANGEN Biotech, Beijing, China). High-throughput sequencing was performed by Novogene Bioinformatics Technology, Co., Ltd. (Beijing, China). Of note, the libraries for the V3-V4 region and full-length sequencing of the 16S rRNA gene were constructed using the Illumina HiSeq2500 and PacBio platforms, respectively. The primers employed in V3-V4 sequencing were: 341F (CCTAYGGGRBGCASCAG) and 806R (GGACTACNNGGGTATCTAAT); in full-length (V1-V9) sequencing were: F: AGAGTTTGATCCTGGCTCAG, R: GNTACCTTGTTACGACTT. PCR amplifications were performed using FastStart High-Fidelity Enzyme Mix and Phusion^®^ High-Fidelity PCR Master Mix (New England Biolabs). PCR conditions were as follows: 98 °C for 30 s, followed by 30 cycles at 94 °C for 45 s, then at 50 °C for 60 s, and finally at 72 °C for 90 s.

### 2.3. Processing of 16S rRNA V3-V4 and Full-Length Sequencing Data

The 16S rRNA V3-V4 and full-length sequencing raw data were processed and analyzed using Quantitative Insights into Microbial Ecology 2020.10 version (QIIME2, 2020.10) [22]: (1) The raw data were quality-controlled using DADA2 [23] to remove chimeric and repetitive redundant sequences. OTUs were selected de novo using 97% (16S rRNA V3-V4) and 99% (16S rRNA full-length) similarity thresholds. (2) The Silva database (silva_132_release) was used to annotate the classification of OTUs. (3) Bacterial alpha diversity was evaluated based on Shannon index and Observed OTUs, and beta diversity was analyzed based on Jaccard, Bray–Curtis, weighted UniFrac, and unweighted UniFrac distance algorithms. Finally, the 16S rRNA full-length sequences were truncated according to the 16S rRNA V3-V4 region PCR primers using usearch11 (http://www.drive5.com/usearch/manual/cmd_search_pcr.html, accessed on 8 July 2022). The resulting 16S rRNA full-length truncated V3-V4 sequences were then analyzed using QIIME2 as described above.

### 2.4. Metagenomic Analysis, Data Processing Assembly, and Metagenome Functional Annotation

After quantification with a Qubit fluorometer, DNA isolated from the same fecal samples were sent to Novogene Bioinformatics Technology, Co., Ltd. (Beijing, China) for metagenomic sequencing on the Illumina NovaSeq 6000 platform with at least 10 G data quantity per sample. Briefly, reads with low-quality bases were removed and host genome sequences were removed using the MOCAT2 software after comparison with the porcine genome reference sequence [24]. After removing host genome sequences, splicing of long reads into contigs was conducted using Megahit v1.2.2 [25] software (parameters: –k-min 21; –k-max 141; –k-step 10; –min-contig-len 500). Gene prediction and construction of nonredundant gene sets from spliced data were conducted using Prodigal software v.2.6.1 [26]. Database alignment and functional annotation were conducted based on the Kyoto Encyclopedia of Genes and Genomes (KEGG) [27], Carbohydrate-Active Enzymes (CAZy) [28] (http://www.cazy.org, accessed on 1 October 2021), and the Comprehensive Antibiotic Resistance (CARD) databases [29] (https://card.mcmaster.ca/, accessed on 1 October 2021).

### 2.5. Statistical Analysis

A Mann–Whitney test and analysis of similarities (ANOSIM) were used for determining the significance analysis of alpha and beta diversity, respectively. Linear discriminant analysis (LDA) effect size (LEfSe) (LDA value > 2) was used for determining between-group differences. R (3.6.0) software was used to draw box plots, principal coordinate analysis (PCoA) plots, and heatmaps using the packages ggplot2 and ggpubr.

## 3. Results

### 3.1. Taxonomic Classification of the Bacteria Using 16S rRNA Genes

**Figure 1:**
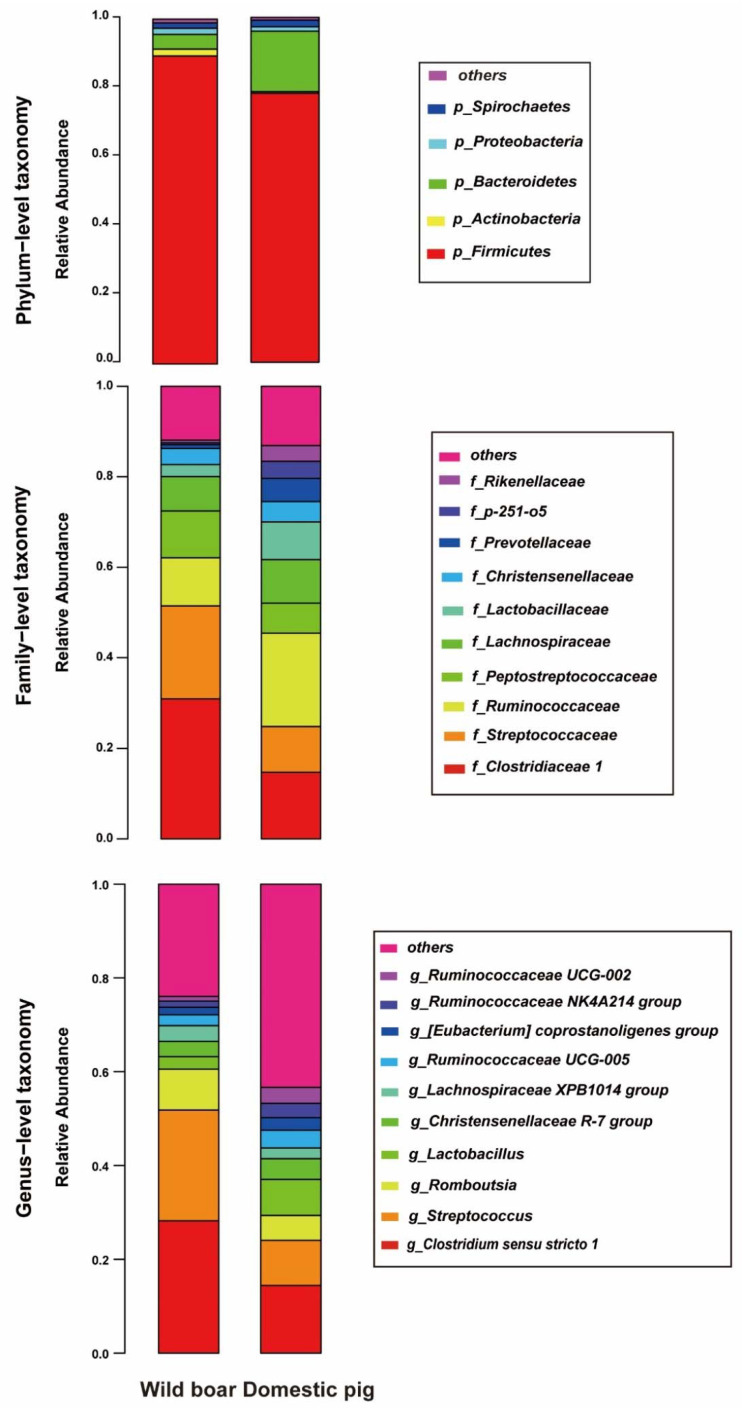
The phylum, family, and genus classification based on 16S rRNA V3-V4 sequences.

A total of 1,158,398 high-quality reads were obtained by sequencing the fecal 16S rRNA V3-V4 of domestic pigs (*n* = 7) and wild boars (*n* = 8) with an average of 77,226 reads per sample, ranging from 62,584 to 85,081; performing OTUs clustering on sequences with a similarity greater than 97% to obtain 27,118 non-redundant OTUs (Table S2, sheet1); and then analyzing their phylum, family, and genus classification levels, as shown in Figure 1. On the other hand, a total of 150,027 high-quality reads were obtained based on 16S rRNA full-length sequencing with an average of 10,001 reads per sample, ranging from 5351 to 30,494, performing OTUs clustering on sequences with a similarity greater than 99% to obtain 58,503 non-redundant OTUs (Table S2, sheet2), and then analyzing the classification level of its phylum, family, genus, and species, as shown in Figure S1. 

The representative sequences numbers of 16S rRNA V3-V4, 16S rRNA full-length, and 16S rRNA full-length truncated V3-V4 analyzed by QIIME2 were 32,991, 106,291, and 487, respectively. Then, the representative sequences of 16S rRNA V3-V4, 16S rRNA full-length, and 16S rRNA full length truncated V3-V4 were aligned at 97% similarity using the software CD-HIT. Among the 32,991 representative sequences of 16S rRNA V3-V4, 25,956 were successfully aligned with 16S rRNA full-length, and 23,289 were successfully aligned with 16S rRNA full-length truncated V3-V4. Among the 487 representative sequences of 16S rRNA full-length truncated V3-V4, 487 were successfully aligned with 16S rRNA full-length.

In 16S rRNA V3-V4 sequencing results, the top 5 relative phylum contents of wild boars and domestic pigs were *Firmicutes*, *Actinobacteria*, *Bacteroidetes*, *Proteobacteria*, and *Spirochaetes*, among which *Firmicutes* had the highest content, accounting for 82.2% of the total average content. Recently, two other studies on wild boars also found *Firmicutes*, *Bacteroidetes*, *Proteobacteria* were the predominant gut microbiota in wild boar bodies [30,31]. Compared with wild boars, *Bacteroidetes* were significantly more prominent in domestic pigs (Whitney test, *p* < 0.01), while *Actinobacteria* were significantly decreased (Whitney test, *p* < 0.01) (Figure 1). In the 16S rRNA full-length sequencing and 16S rRNA full-length truncated V3-V4 results, *Firmicutes*, *Spirochaetes*, and *Bacteroidetes* also ranked in the top 5 in relative content (Figures S1 and S2). Among them, *Firmicutes* and *Spirochaetes* showed consistent results in the three groups. However, the content of *Bacteroidetes* in domestic pigs was significantly higher than that in wild boars in the 16S rRNA V3-V4 sequencing results (Whitney test, *p* < 0.01), whereas there was no significant difference in the other two groups (Figure S3).

At the family level, *Clostridiaceae 1*, *Streptococcaceae*, and *Ruminococcaceae* were the three most dominant bacterial groups in wild boars and domestic pigs, accounting for 53.76% of the total average content. Compared with wild boars, the abundances of *Ruminococcaceae* and *Lactobacillaceae* were significantly higher in domestic pigs, while *Clostridiaceae1* was significantly lower (Whitney U-test, *p* < 0.01) (Figure 1). Consistent with the 16S rRNA full-length sequencing and 16S rRNA full-length truncated V3-V4 results, the relative contents of *Ruminococcaceae*, *Streptococcaceae*, *Lachnospiraceae*, *Lactobacillaceae*, *Peptostreptococcaceae*, *Clostridiaceae 1*, *Prevotellaceae* also ranked in the top 10 (Figures S1 and S2). Four of the seven shared bacteria (*Streptococcaceae*, *Lachnospiraceae*, *Lactobacillaceae*, *Peptostreptococcaceae*) showed consistent results. However, in the 16S rRNA V3-V4 sequencing results, *Clostridiaceae1* was significantly increased in wild boars, while the contents of *Prevotellaceae* and *Ruminococcaceae* were significantly increased in domestic pigs (Whitney test, *p* < 0.01), but the results of the other two groups did not change significantly (Figure S4). This was similar to the results of another study which found that African warthogs had lower levels of *Ruminococcaceae* and *Prevotellaceae* compared with domestic pigs on traditional commercial farms [12].

At the genus level (Figure 1), the top 2 highest relative content of wild boars and domestic pigs were *Clostridium sensu stricto 1*, and *Streptococcus*, accounting for about 37.93% of the total average content. Consistent with the 16S rRNA full-length sequencing and 16S rRNA full-length truncated V3-V4 results, the relative content of *Streptococcus*, *Christensenellaceae R-7 group*, *Clostridium sensu stricto 1*, and *Lactobacillus* also ranked in the top 10 (Figures S1 and S2). Three of the four shared bacteria (*Streptococcus*, *Christensenellaceae R-7 group*, *Lactobacillus*) showed consistent results. However, in the 16S rRNA V3-V4 sequencing results, *Clostridium sensu stricto 1* was significantly increased in wild boars (Whitney test, *p* < 0.01), but the results of the other two groups did not change significantly (Figure S5).

In the results of phylum, family, and genus content examination, we found that the results of 16S rRNA full-length sequencing and 16S rRNA full-length truncated V3-V4 showed a consistent trend of shared bacterial microbiota, while the results of 16S rRNA V3-V4 sequencing were mostly consistent with the other two groups with only a few inconsistencies. The main reason for this difference may be the difference between the two sequencing technologies rather than the detection region. In another comparison of the sequencing results of V3-V4 short read and 16S full-length-based synthetic long read, it was also found that most of the gut microbiota at the phylum, family and genus levels had relatively high similarity [21].

### 3.2. Alpha and Beta Diversities of Gut Microbiota

**Figure 2:**
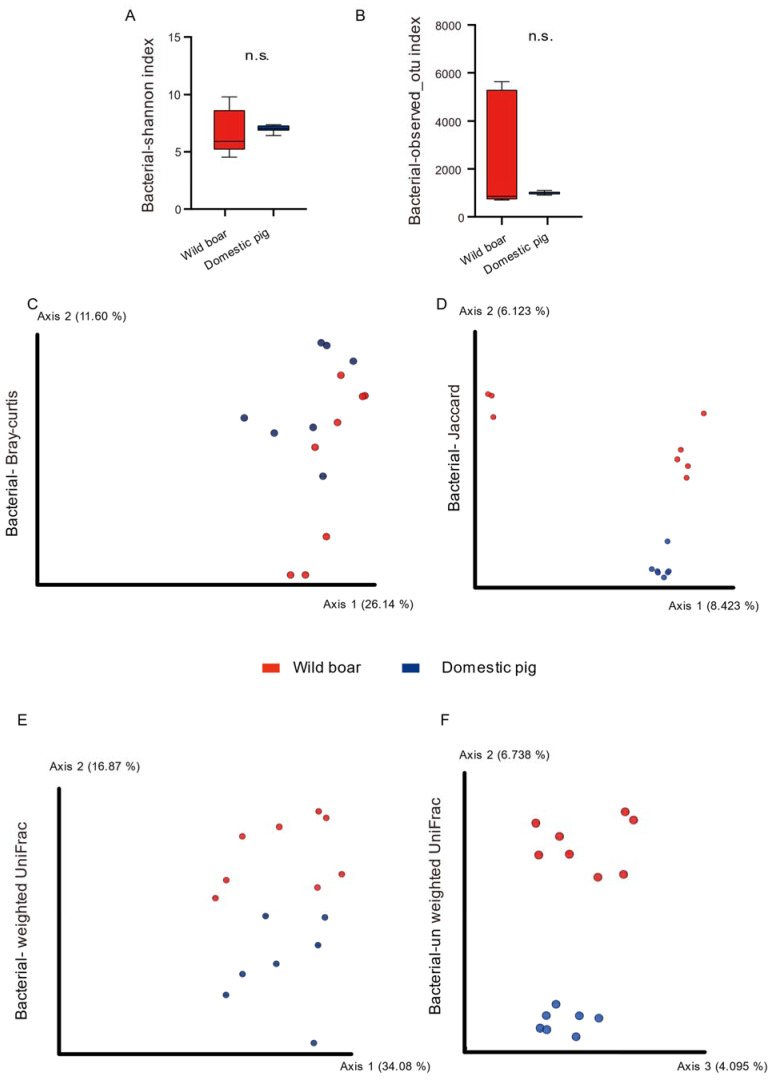
The alpha and beta diversity of pigs’ gut microbiota based on 16S rRNA V3-V4 sequences. Alpha diversity includes Shannon Index (**A**) and Observed OTUs. (**B**): The red and blue bars represent wild boars and domestic pigs, respectively. n.s., no significance, Mann–Whitney U-test. (**C**): PCoA of bacterial Bray–Curtis. (**D**): PCoA of bacterial Jaccard. (**E**): PCoA of bacterial weighted UniFrac. (**F**): PCoA of bacterial unweighted UniFrac. ANOSIM, *p* < 0.05, the red and blue circles represent the bacterial communities of the wild boars and domestic pigs, respectively.

Figure 2 shows the alpha and beta diversity of the 16S rRNA V3-V4 diversity. There was no significant difference in alpha diversity between wild boars and domestic pigs (Figure 2A,B, n.s., Mann–Whitney U-test, Table S2, sheet3). In the beta diversity (Bray–Curtis, Jaccard, weighted UniFrac, unweighted UniFrac) analysis, we found that domestic pigs were significantly different from wild boars (Figure 2C–F, ANOSIM, *p* < 0.05, Table S2, sheet3) (16S rRNA full-length sequencing had same results in beta diversity and alpha diversity, Figure S6, Table S2, sheet3). Four measurements were found to be significantly different between domestic and wild boars, indicating significant differences in the composition, abundance, and phylogeny of the microbiota between the two groups.

### 3.3. Differences in Bacterial Communities between Wild Boars and Domestic pigs

**Figure 3:**
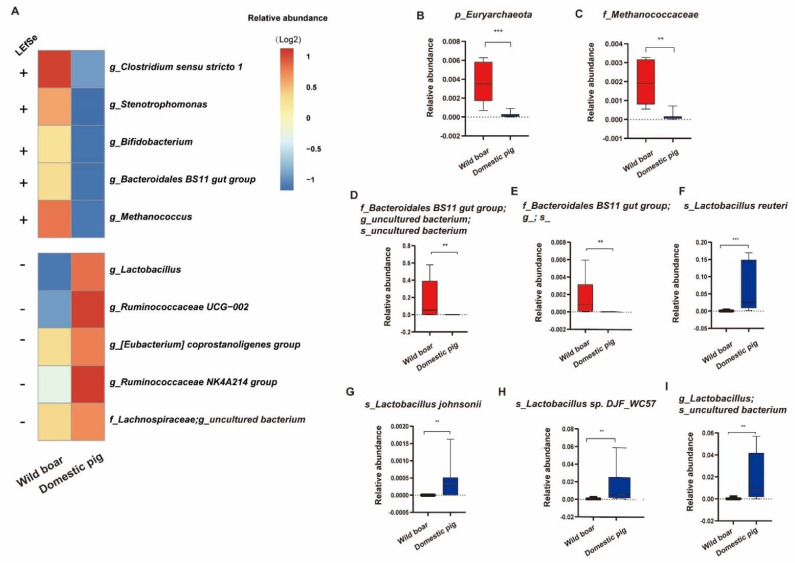
Differences in bacterial communities between wild boars and domestic pigs. (**A**): The average relative abundance of the LEfSe-identified bacterial taxa in the wild boars and domestic pigs is plotted as a heatmap. “+” indicates significantly high abundance in wild boars, “-” indicates significantly high abundance in domestic pigs (*p* < 0.05, LDA cutoff = 2.0). Differential gut microbiota at the genus level: (**B**): *p_Euryarchaeota*, (**C**): *f_Methanococcaceae*. (**D**–**I**): Differential gut microbiota at species level: (**D**): *f_Bacteroidales BS11 gut group*; *g_uncultured bacterium*; *s_uncultured bacterium*, (**E**): *f_Bacteroidales BS11 gut group*; g_; s_, (**F**): s*_Lactobacillus reuteri*, (**G**): *s_Lactobacillus johnsonii*, (**H**): *s_Lactobacillus sp.DJ_WC57*, (**I**): *g_Lactobacillus; s_uncultured bacterium*, Whitney U-test, ** *p* < 0.01, *** *p* < 0.001. The red bars and blue bars represent wild boars and domestic pigs. The line in the box represents the middle value, and the error bars represented the lowest and highest values, respectively.

LEfSe analysis was conducted based on the genus level at a relative abundance of at least 0.1% to identify specific bacterial taxa in both the wild boars and domestic pigs (Figure 3). At the genus level (Figure 3A), 5 taxa (e.g., *g_Clostridium sensu stricto 1*, *g_Bifidobacterium*, *g_Bacteroidales BS11 gut group*, and *g_Methanococcus*, etc. *p* < 0.05, LDA cutoff = 2.0) were significantly increased in wild boars, while 5 taxa (e.g., *g_Lactobacillus*, g_*Ruminococcaceae UCG-010*, *f_Lachnospiraceae; g_uncultured bacterium*, etc. *p* < 0.05, LDA cutoff = 2.0) were significantly increased in domestic pigs. Interestingly, we found that *p_Euryarchaeota* and *f_Methanococcaceae* were also significantly higher in wild boars than in domestic pigs.

At the species level (Figure 3D–I), we also found that the abundance of *f_Bacteroidales BS11 gut group*; *g_uncultured bacterium; s_uncultured bacterium* and *f_Bacteroidales BS11 gut group*; g_; s_ were significantly increased in wild boars, while the abundance of *s_Lactobacillus reuteri*, *s_Lactobacillus johnsonii*, *s_Lactobacillus sp.DJF_WC57*, and *g_Lactobacillus*; *s_uncultured bacterium* were significantly increased in domestic pigs (Whitney U-test, *p* < 0.05).

### 3.4. Top 10 KEGG Categories and Differential KEGG Categories

Shotgun metagenomics sequencing on the Illumina NovaSeq 6000 platform generated a total of 1,287,541,224 paired-end raw sequences (raw reads). 1,245,613,476 high-quality sequences (clean reads) were obtained after removing low-quality and contaminated sequences. After stitching pairs of high-quality sequences based on the overlapping regions, 13,701,836 long reads (contigs) (ranging between 454,042–1,112,050) were generated, and the number of genes (ORFs) obtained was 28,419,118 (Table S3). 

**Figure 4:**
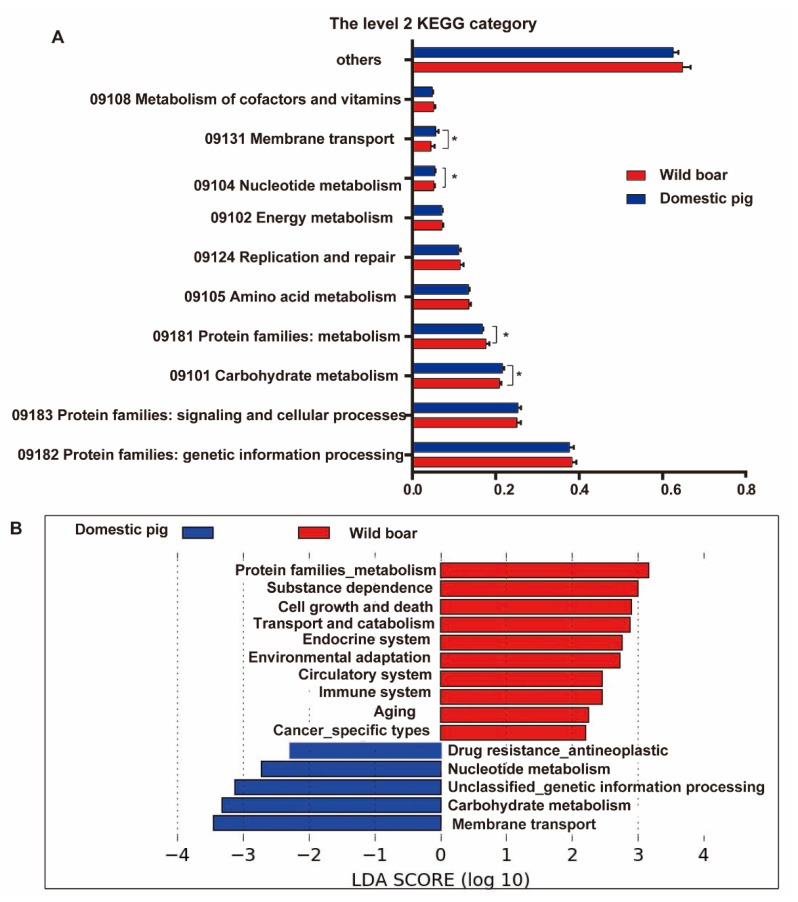
Top 10 KEGG categories and the LEfSe analysis between wild boars and domestic pigs. (**A**): Top 10 composition of KEGG category. The red bars and blue bars represent wild boars and domestic pigs, respectively. Whitney U-test, * *p* < 0.05. (**B**): The LEfSe analysis between wild boars and domestic pigs. The red bars and blue bars represent wild boars and domestic pigs, respectively. *p* < 0.05, LDA cutoff = 2.0.

Based on the composition analysis of the KEGG category, we obtained the top 10 KEGG categories of average relative contents (Figure 4A): “genetic information processing of protein families”, “signal and cellular process of protein families”, and “carbohydrate metabolism” were the three metabolic pathways with the highest relative contents, about 39.75% of the total contents. Compared with wild boars, “membrane transport”, “nucleoside metabolism”, and “carbohydrate metabolism” of domestic pigs were significantly increased, while “protein families: metabolism” was significantly reduced.

Figure 4B refers to the differential KEGG categories. Of these, 10 KEGG categories (e.g., “environmental adaptation”, “transport and catalysis”, “circulation system”, “cell growth and death”, “immune system”, “endocrine system”, etc. *p* < 0.05, LDA cutoff = 2.0) were significantly increased in the wild boars, while 5 KEGG categories (e.g., “carbohydrate metabolism”, “nucleoside metabolism”, “drug resistance antineoplastic”, “membrane transport”, etc. *p* < 0.05, LDA cutoff = 2.0) were significantly increased in the domestic pigs.

### 3.5. Diversity and Differential KEGG Pathways

**Figure 5:**
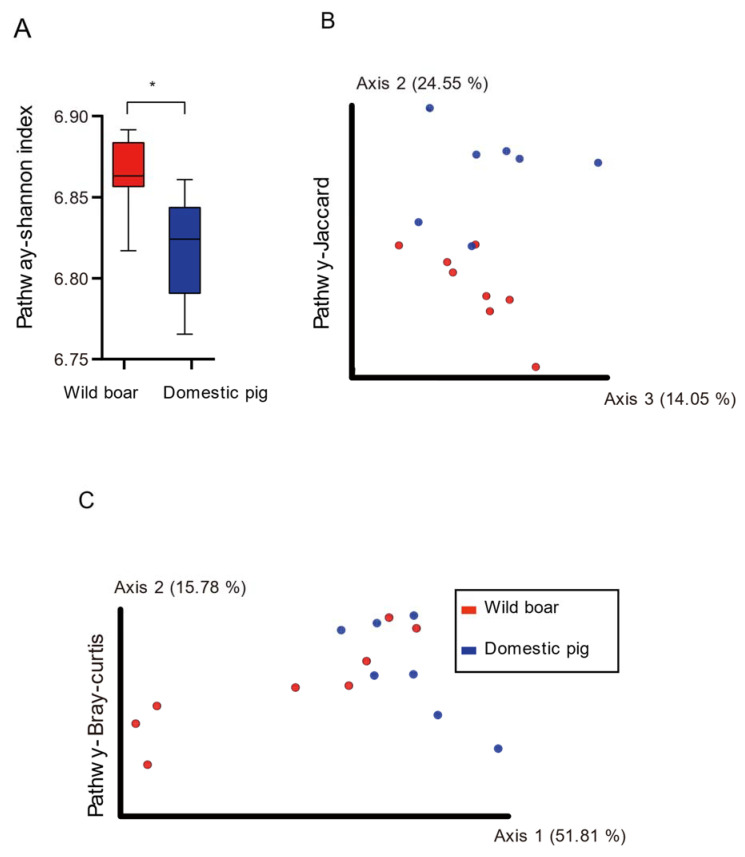
The alpha and beta diversity of KEGG pathways between wild boars and domestic pig Alpha diversity in Shannon index (**A**), * *p* < 0.05, Mann–Whitney U-test. Red and blue bars represent wild boars and domestic pigs, respectively. (**B**): Principal Coordinate Analysis (PCoA) of pathway based on Jaccard distances. (**C**): Principal Coordinate Analysis (PcoA) of pathway based on Bray–Curtis distances. ANOSIM, *p* < 0.05, red, blue circles represent bacterial communities of pigs in the wild boars and domestic pigs, respectively.

In the analysis of alpha diversity, compared with wild boars, the Shannon index of the pathway of domestic pigs decreased significantly (Figure 5A, Mann–Whitney test, *p* < 0.05, Table S2, sheet3). In the Bray–Curtis and Jaccard analyses of KEGG pathways, we found that wild boars and domestic pigs were significantly different (Figure 5B,C, ANOSIM, *p* < 0.05, Table S2, sheet3). (KOs also had significant differences in beta diversity, ANOSIM, *p* < 0.05, Table S2, sheet3, as shown in Figure S7).

**Figure 6:**
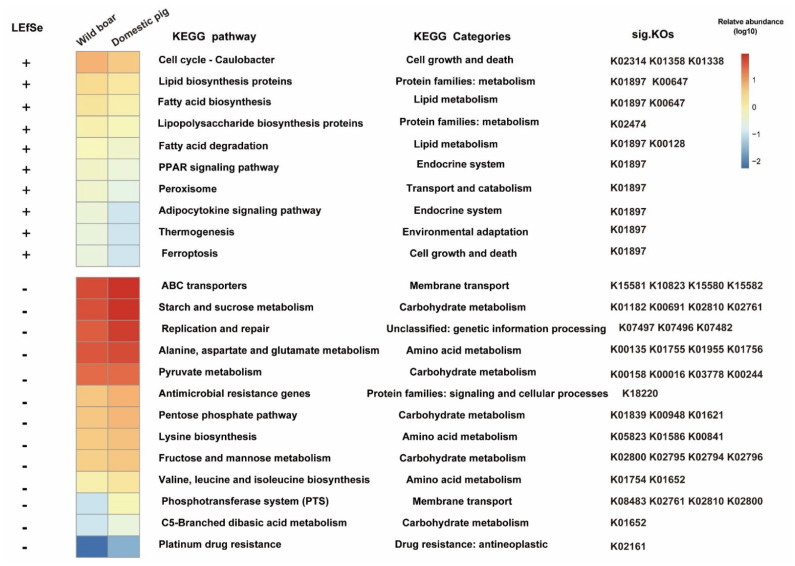
KEGG pathways found to be significantly associated with wild boars and domestic pigs. The average relative abundance of the LEfSe-identified KEGG pathways in the wild boar and domestic pig were plotted as a heatmap. “+” indicates significantly high abundance in wild boar, “-” indicates significantly high abundance in domestic pig (*p* < 0.05, LDA cutoff = 2.0). The corresponding KEGG categories and corresponding differential KOs of KEGG pathway are shown on the left.

Figure 6 shows the differential KEGG pathway identified by LefSe in wild boars and domestic pigs, and its corresponding KEGG Categories and differential KOs. The “fatty acid degradation”, “fatty acid biosynthesis”, “lipid biosynthesis proteins”, “lipopolysaccharide biosynthesis proteins”, “peroxisome”, “thermogenesis”, etc., were significantly increased in wild boars, while the “starch and sucrose metabolism”, “pyruvate metabolism”, “pentose phosphate pathway”, “alanine, aspartate and glutamate metabolism”, “lysine biosynthesis”, “valine leucine and isoleucine biosynthesis”, “antimicrobial resistance genes”, etc., were significantly increased in domestic pigs (Figure 6).

### 3.6. CAZymes Diversity and the Changes of CAZymes between Wild Boar Domestic Pig

**Figure 7:**
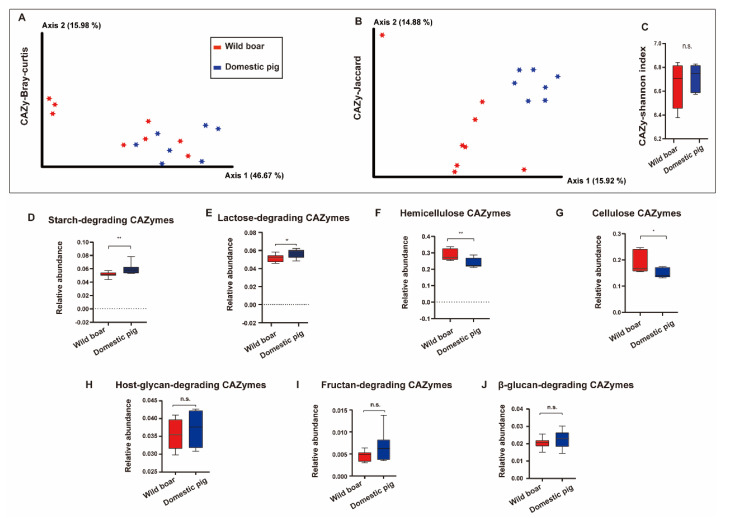
Diversity of CAZymes and the change of CAZymes between wild boar and domestic pig. (**A**–**C**): The CAZy of beta and alpha diversity. (**A**): Principal Coordinate Analysis (PCoA) of CAZymes based on Bray–Curtis distances. (**B**): Principal Coordinate Analysis (PCoA) of CAZymes based on Jaccard distances. ANOSIM, *p* < 0.05, red and blue circles represent bacterial communities in pigs in the wild boar and domestic pig groups, respectively. Alpha diversity is Shannon index (c), n.s., no significance, Mann–Whitney U-test. Red and blue bars represent wild boar and domestic pigs, respectively. Different CAZymes of wild boar between domestic pigs. (**D**): Starch-degrading CAZymes, (**E**): Lactose-degrading CAZymes, (**F**): Hemicellulose CAZymes, (**G**): Cellulose CAZymes, (**H**): Host-glycan-degrading CAZymes, (**I**): Fructan-degrading CAZymes, (**J**): β-glucan-degrading CAZymes. Whitney U-test, * *p* <0.05, ** *p* <0.01. The red bars and blue bars represent wild boar and domestic pigs, respectively. Boxplot indicates the interquartile range (IQR). The line in the box represents the middle value, and the error bars represent the lowest and highest values, respectively.

Similar to the KEGG database analysis results, in the analysis of beta diversity (Bray–Curtis and Jaccard) of CAZymes, we found significant differences between wild boars and domestic pigs (Figure 7A, B, ANOSIM, *p* < 0.05, Table S2, sheet3). In the analysis of alpha diversity, the Shannon index of CAZymes was not significantly changed between wild boars and domestic pigs (Figure 7C, Mann–Whitney test, n.s., Table S2, sheet3).

Compared with wild boars, we found that the relative contents of starch-degrading CAZymes and lactose-degrading CAZymes were significantly increased in domestic pigs, while the relative contents of cellulose CAZymes and hemicellulose CAZymes were significantly decreased (Figure 7D–G, Mann–Whitney test, *p* < 0.05). (KOs had similar results, alpha-amylase-related KOs were increased in domestic pigs, while KOs related to endo-1,4-beta-xylanase were significantly increased in wild boar, Mann–Whitney test, *p* < 0.05, as shown in Figure S7.) The host-glycan-degrading CAZymes, Fructan-degrading CAZymes, and β-glucan-degrading CAZymes in both groups were not significantly changed during evolution (Figure 7H–J).

### 3.7. The Analysis of Antibiotic Resistance Genes (ARGs) between Domestic and Wild Boars

**Figure 8:**
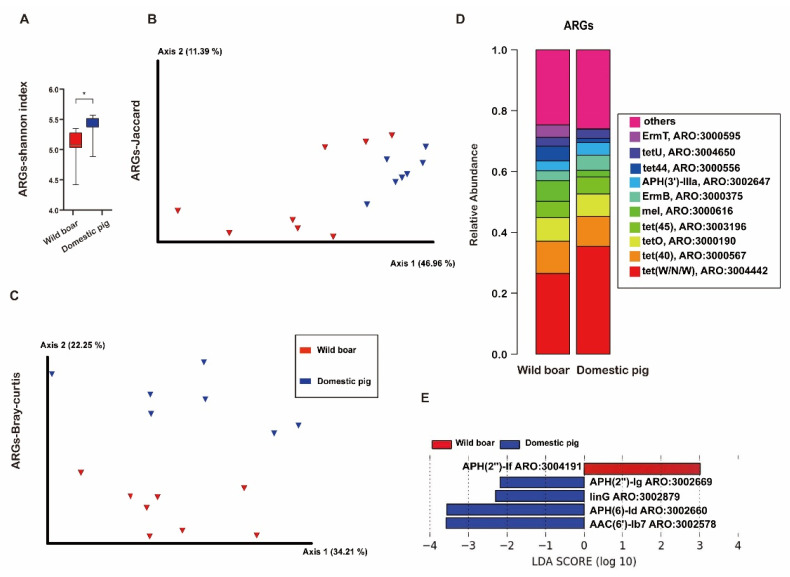
The comparison of ARGs between domestic pigs and wild boars. Alpha diversity is Shannon index (**A**), * *p* < 0.05, Mann–Whitney U-test. Red and blue bars represent wild boar and domestic pigs, respectively. (**B**): Principal Coordinate Analysis (PCoA) of ARGs based on Jaccard distances. (**C**): Principal Coordinate Analysis (PCoA) of ARGs based on Bray–Curtis distances. ANOSIM, *p* < 0.05, red and blue circles represent bacterial communities of pigs in the wild boar and domestic pig groups, respectively. (**D**): Top10 ARGs classification of domestic pigs and wild boars. (**E**): The LEfSe analysis of ARGs between wild boars and domestic pigs.

Figure 8 shows the comparison of ARGs between domestic pig and wild boar.

The Shannon index of ARGs of domestic pigs was significantly higher than that of wild boars (Figure 8A, Mann–Whitney test, *p* < 0.05, Table S2, sheet3). Bray–Curtis and Jaccard of ARGs of domestic pig and wild boar were significantly different (Figure 8B,C, ANOSIM, *p* < 0.05, Table S2, sheet3).

The top 10 ARGs in wild boars and domestic pigs were tet(W/N/W) (ARO:3004442), tet(40)(ARO:3000567), tetO(ARO:3000190), tet(45)(ARO:3003196), mel(ARO:3000616), ErmB(ARO:3000375), APH(3′)-IIIa(ARO:3002647), tet44(ARO:3000556), tetU(ARO:3004650) and ErmT(ARO:3000595), respectively. Interestingly, six of them belong to tetracycline antibiotic, accounting for about 60.3% of the total average relative content, including the ARGs of top3(tet (w/N/W), tet (40), and tetO) (Figure 8D).

LEfSe analysis found that APH(″)-If(ARO:3004191) were significantly increased in the wild boars, while 4 ARGs, (APH(2″)-Ig(ARO:3002669), linG(ARO:3002879), APH(6)-Id(ARO:3002660), and AAC(6′)-Ib7(ARO:3002578) were significantly increased in the domestic pigs (*p* < 0.05, LDA cutoff = 2.0, Figure 8E).

## 4. Discussion

The survival and reproduction of an organism derives from its capability to adjust its growth, development, metabolism, and behavior to changing environments. Since wild boars live in the wild and live on leaves, grass, roots, berries, and insects, they have strong environmental adaptability, disease resistance, and rough feeding resistance. However, after a long period of domestication, modern domestic pigs mainly feed upon grain-based diets and have strong digestive, growth, and reproductive performance. Interestingly, in this study, we found that the composition and function of gut microbiota in wild pigs and domestic pigs have also changed over the long evolution.

In this experiment, we found that the abundance of *Lactobacillus* was significantly increased in domestic pigs and *Bifidobacterium* was significantly increased in wild boar, which was consistent with the previous report that wild boars were abundant in *Bifidobacterium*, while domesticated animals become *lactobacillus* [32,33]. At the same time, we also found that four highly abundant *Lactobacillus* species *(s_Lactobacillus reuteri*, *s_Lactobacillus johnsonii*, *s_Lactobacillus sp.DJF_WC5*, and *g_Lactobacillus;s_uncultured bacterium*) were significantly increased in domestic pigs, indicating that some gut microbiota has changed during the long domestication process. *Lactobacillus* is significantly more abundant in domestic pigs, which may be related to its high digestibility. Studies have found that adding *Lactobacillus* to the diet can promote the digestion and absorption of nutrients in animals and improve animal growth performance and feed conversion efficiency [34,35]. *Bifidobacterium* is significantly more abundant in wild boars, which may be related to their disease resistance. *Bifidobacterium* can inhibit the invasion of intestinal pathogens and promote the healthy growth of the body [36,37]. However, the reason for the difference between these two strains in the two groups of pigs may be due to the genetic evolution of the organism and modern artificial rearing.

In addition, we also found a similar phenomenon in KEGG metabolic functions of gut microbiota. The relative abundances of metabolic pathways such as “environmental adaptation”, “immune system”, “fatty acid degradation”, and “fatty acid biosynthesis” were significantly increased in wild boar feces, which may be associated with environmental adaptation, disease resistance, and immune function in wild boars. In another study on wild boars and domestic pigs, it was also found that “fatty acid degradation”, and “fatty acid biosynthesis” were significantly more prominent in wild boars [38]. Studies have shown that short-chain fatty acids (SCFAs) can affect gastrointestinal motility, maintain intestinal barrier integrity, reduce inflammation, improve autoimmune diseases and allergies, and promote the health of the body by resisting the invasion of pathogenic bacteria [39,40,41]. On the other hand, we found that the “carbohydrate metabolism”, “starch and sucrose metabolism”, and the starch degradation CAZymes were significantly more prominent in domestic pigs, while the cellulose and hemicellulose degradation CAZymes and endo-1,4-beta-xylanase-related KOs were significantly more prominent in wild boars, which may be related to the high feed collection rate of domestic pigs and the coarse feeding tolerance of wild boar. Meanwhile, we also found that “valine leucine and isoleucine biosynthesis”, “lysine biosynthesis”, and “alanine, aspartate and glutamate metabolism” were significantly increased in domestic pigs. These amino acids can improve the growth performance and reproductive performance of pigs, and studies have found that the addition of these amino acids to diets can improve piglet nutrient metabolism and daily average weight gain; maintain the integrity of the intestinal barrier; and improve the body’s immune system [42,43,44,45].

Interestingly, we found that the content of archaeal *p_Euryarchaeota*, *f_Methanococcaceae,* and *g_Methanococcus* decreased significantly during pig evolution. Archaea are considered to be an important part of the host’s microbiome, mainly living in extreme environments, such as volcanoes, salt lakes, and the guts of animals and humans [46]. *Methanococcuceae* specifically metabolize hydrogen derived from anaerobic fermentation of carbohydrates to methane [47] and may be involved in disease-related processes [46]. It is found that the diversity of archaea in great apes of human ancestors was higher than that in humans, which indicates that archaea content has declined during human evolution [48]. Compared with wild boars, the content of *p_Euryarchaeota*, *f_Methanococcaceae,* and *g_Methanococcus* in domestic pigs was significantly lower, possibly due to environment, diet, and host genes altered during the long evolutionary process, thus allowing the gut microbiota to change due to host-selective pressure [49,50,51,52,53].

Notably, although none of the experimental animals used antibiotics, analysis of resistance genes revealed that the diversity of ARGs, “antimicrobial resistance genes”, and “drug resistance antineoplastic” was significantly higher in domestic pigs than in wild boars, which may be mainly due to the antibiotic exposure and environmental pollution associated with the artificial husbandry [54]. A similar phenomenon was detected by Guo et al., both in the wild and in captive pandas, where the gut microbiota of captive pandas contained a higher abundance of antibiotic resistance genes, heavy metal tolerance genes, and virulence factor genes, suggesting that artificial feeding management and environment negatively affect the gut microbiota of pandas [55]. On the other hand, we found that the most abundant ARGs in wild boars and domestic pigs belong to tetracycline antibiotics, which were commonly used antibiotics in livestock farming. The tetracycline resistance gene may be located on a mobile genetic element, allowing it to be widely transferred and spread throughout the environment [56]. It was found that agricultural activities and urban sewage activities in rural areas were the most important reasons for the widespread existence of tetracycline resistance genes and mobile genes [57].

## 5. Conclusions

In this study, *Bifidobacterium* and *Methanococcaceae* were found to be significantly more abundant in wild boars, while *Lactobacillus* was significantly more abundant in domestic pigs after domestication. In addition, the functions of the gut microbiota also changed similarly with host domestication, with wild boars being significantly increased in “environmental adaptation”, “immune system”, “fatty acid degradation and biosynthesis”, and cellulose-hemicellulose degradation CAZymes, whereas domestic pigs were significantly increased in starch degradation pathways and CAZymes, as well as some amino acid pathways related to growth and reproductive performance. In addition, the diversity of ARGs and “antimicrobial resistance genes” in domestic pigs also increased significantly. In conclusion, our data show that the composition and function of the pig gut microbiota have changed significantly after domestication, which provides some data on the evolution of the gut microbiota.

## Data Availability

The sequencing data were deposited in the NCBI GenBank Sequence Read Archive (SRA) under BioProject numbers PRJNA843193 and PRJNA844176 (SRA, http://www.ncbi.nlm.nih.gov/sra, accessed on 1 October 2021).

## Supplementary Materials

The following supporting information can be downloaded at: https://www.mdpi.com/article/10.3390/ani12172270/s1. Figure S1: The phylum, family, genus, and species classification based on 16S rRNA full-length sequences; Figure S2: The phylum, family, genus, and species classification based on 16S rRNA full-length truncated V3-V4 sequences; Figure S3: Phylum-level shared bacteria between 16S rRNA V3-V4, 16S rRNA full-length and 16S rRNA full-length truncated V3-V4 results; Figure S4: Family-level shared bacteria between 16S rRNA V3-V4, 16S rRNA full-length and 16S rRNA full-length truncated V3-V4 results; Figure S5: Genus-level shared bacteria between 16S rRNA V3-V4, 16S rRNA full-length and 16S rRNA full length truncated V3-V4 results; Figure S6: The diversity of pigs’ gut microbiota based on 16S rRNA full-length sequences; Figure S7: Diversity of KOs and differences of KOs related to starch, cellulose, and hemicellulose between wild boar domestic pig; Table S1: Dietary ingredient composition and nutrient levels (%); Table S2: *Sheet1*: Sample metadata and 16S rRNA V3-V4 non-redundant OTUs table summary; *Sheet2*: Sample metadata and 16S rRNA full-length non-redundant OTUs table summary; *Sheet3*: The statistical analysis results for Figure 2, Figure 5, Figure 7 and Figure 8, Figures S6 and S7; Table S3: Summary of whole-genome shotgun (WGS) sequencing.
