# Supplementary material for: Comparison of Changes in Gut Microbiota in Wild Boars and Domestic Pigs Using 16S rRNA Gene and Metagenomics Sequencing Technologies
Source: Animals (Basel). 2022 Sep 1;12(17):2270. doi: 10.3390/ani12172270 (PMC9454828; doi:10.3390/ani12172270)
Supplement: Supplementary file 1 [file animals-12-02270-s001.zip › animals-1802100-Supplementary/Fig.S2.pdf]

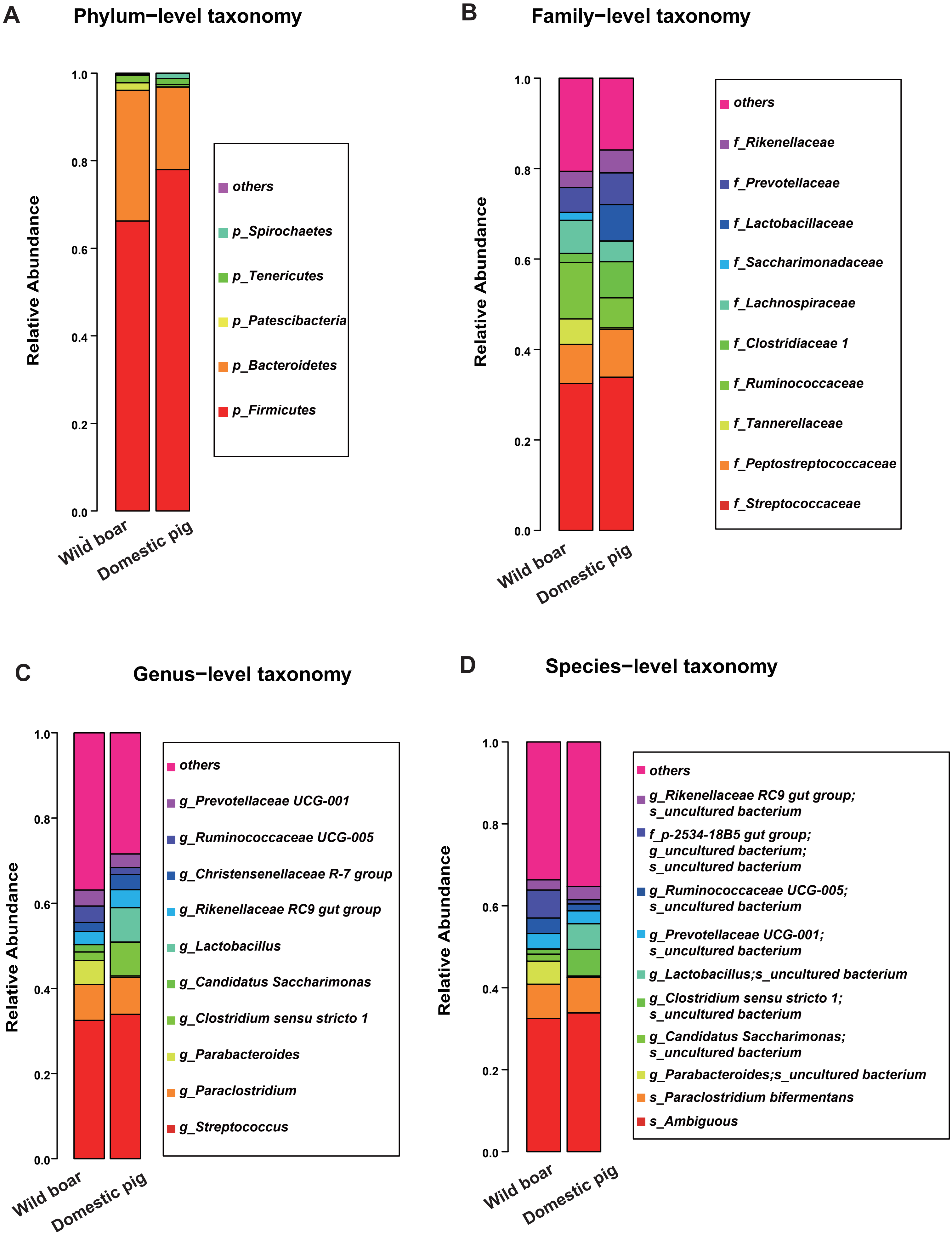

A
Phylum−level taxonomy
Relative Abundance
0.0
0.2
0.4
0.6
0.8
1.0
Wild boar
Domestic pig
others
p_Spirochaetes
p_Tenericutes
p_Patescibacteria
p_Bacteroidetes
p_Firmicutes
B
Family−level taxonomy
Relative Abundance
0.0
0.2
0.4
0.6
0.8
1.0
Wild boar
Domestic pig
others
f_Rikenellaceae
f_Prevotellaceae
f_Lactobacillaceae
f_Saccharimonadaceae
f_Lachnospiraceae
f_Clostridiaceae 1
f_Ruminococcaceae
f_Tannerellaceae
f_Peptostreptococcaceae
f_Streptococcaceae
C
Genus−level taxonomy
Relative Abundance
0.0
0.2
0.4
0.6
0.8
1.0
Wild boar
Domestic pig
others
g_Prevotellaceae UCG-001
g_Ruminococcaceae UCG-005
g_Christensenellaceae R-7 group
g_Rikenellaceae RC9 gut group
g_Lactobacillus
g_Candidatus Saccharimonas
g_Clostridium sensu stricto 1
g_Parabacteroides
g_Paraclostridium
g_Streptococcus
D
Species−level taxonomy
Relative Abundance
0.0
0.2
0.4
0.6
0.8
1.0
Wild boar
Domestic pig
others
g_Rikenellaceae RC9 gut group; s_uncultured bacterium
f_p-2534-18B5 gut group; g_uncultured bacterium; s_uncultured bacterium
g_Ruminococcaceae UCG-005; s_uncultured bacterium
g_Prevotellaceae UCG-001; s_uncultured bacterium
g_Lactobacillus;s_uncultured bacterium
g_Clostridium sensu stricto 1; s_uncultured bacterium
g_Candidatus Saccharimonas; s_uncultured bacterium
g_Parabacteroides;s_uncultured bacterium
s_Paraclostridium bifermentans
s_Ambiguous
