# Supplementary material for: Comparison of Changes in Gut Microbiota in Wild Boars and Domestic Pigs Using 16S rRNA Gene and Metagenomics Sequencing Technologies
Source: Animals (Basel). 2022 Sep 1;12(17):2270. doi: 10.3390/ani12172270 (PMC9454828; doi:10.3390/ani12172270)
Supplement: Supplementary file 1 [file animals-12-02270-s001.zip › animals-1802100-Supplementary/Fig.S3.pdf]

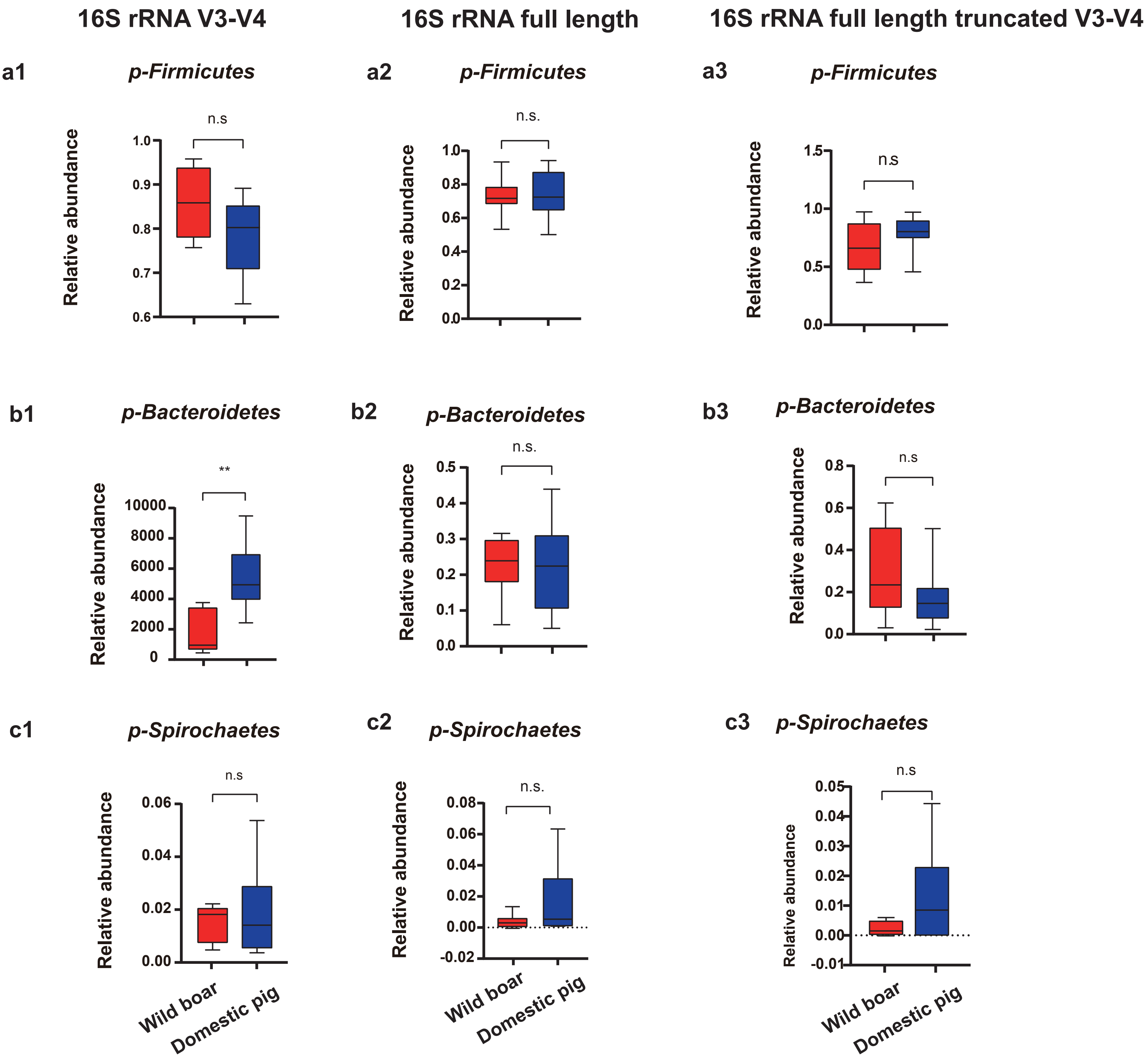
16S rRNA V3-V4
16S rRNA full length
16S rRNA full length truncated V3-V4
a1
p-Firmicutes
n.s
a2
p-Firmicutes
n.s.
a3
p-Firmicutes
n.s
b1
p-Bacteroidetes
**
b2
p-Bacteroidetes
n.s.
b3
p-Bacteroidetes
n.s
c1
p-Spirochaetes
n.s
c2
p-Spirochaetes
n.s.
c3
p-Spirochaetes
n.s
Relative abundance
Wild boar
Domestic pig
