# Supplementary material for: Comparison of Changes in Gut Microbiota in Wild Boars and Domestic Pigs Using 16S rRNA Gene and Metagenomics Sequencing Technologies
Source: Animals (Basel). 2022 Sep 1;12(17):2270. doi: 10.3390/ani12172270 (PMC9454828; doi:10.3390/ani12172270)
Supplement: Supplementary file 1 [file animals-12-02270-s001.zip › animals-1802100-Supplementary/Fig.S4.pdf]

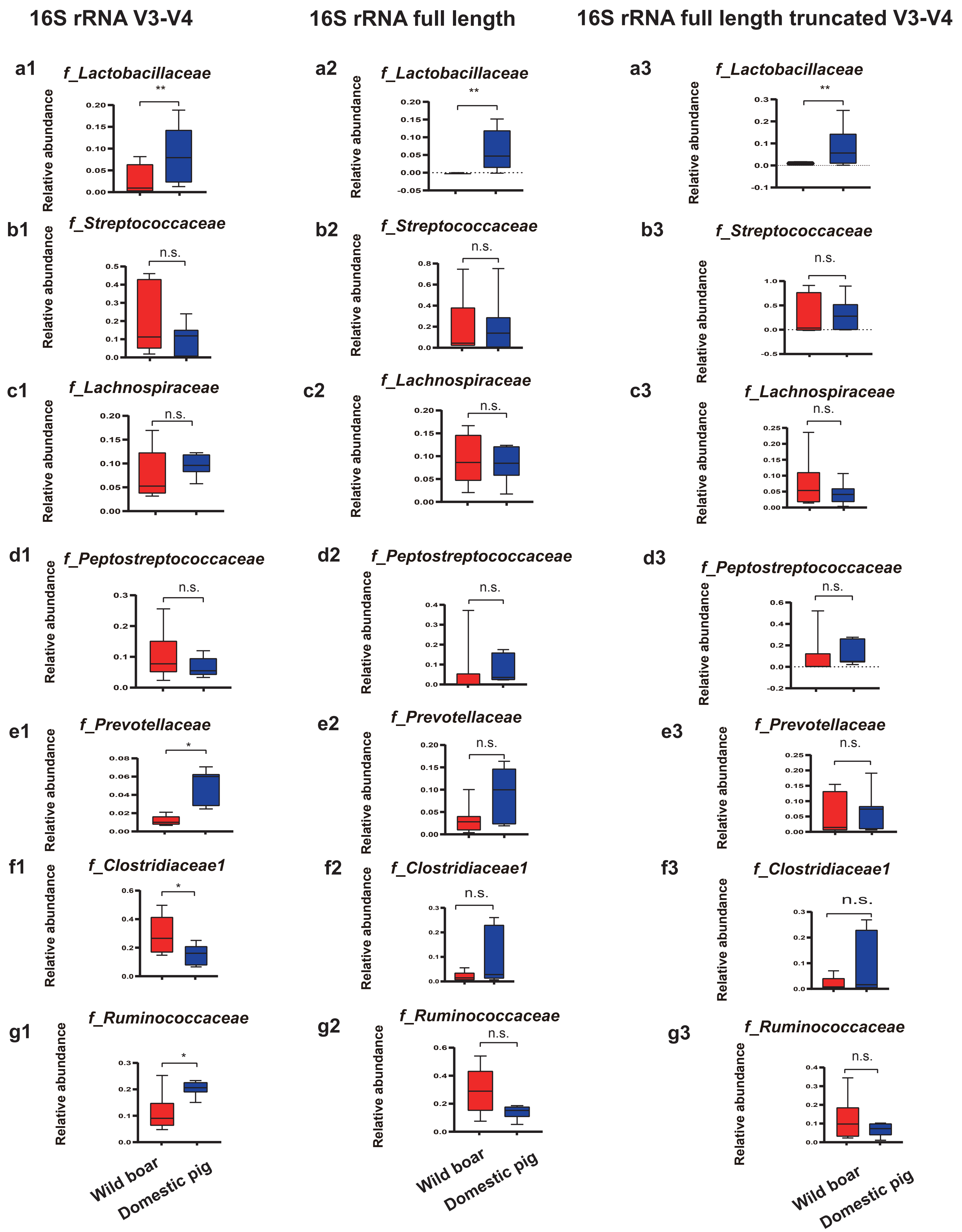

16S rRNA V3-V4
16S rRNA full length
16S rRNA full length truncated V3-V4
a1
f_Lactobacillaceae
a2
a3
b1
f_Streptococcaceae
b2
b3
c1
f_Lachnospiraceae
c2
c3
d1
f_Peptostreptococcaceae
d2
d3
e1
f_Prevotellaceae
e2
e3
f1
f_Clostridiaceae1
f2
f3
g1
f_Ruminococcaceae
g2
g3
Relative abundance
**
n.s.
*
Wild boar
Domestic pig
