# Supplementary material for: Comparison of Changes in Gut Microbiota in Wild Boars and Domestic Pigs Using 16S rRNA Gene and Metagenomics Sequencing Technologies
Source: Animals (Basel). 2022 Sep 1;12(17):2270. doi: 10.3390/ani12172270 (PMC9454828; doi:10.3390/ani12172270)
Supplement: Supplementary file 1 [file animals-12-02270-s001.zip › animals-1802100-Supplementary/Fig.S5.pdf]

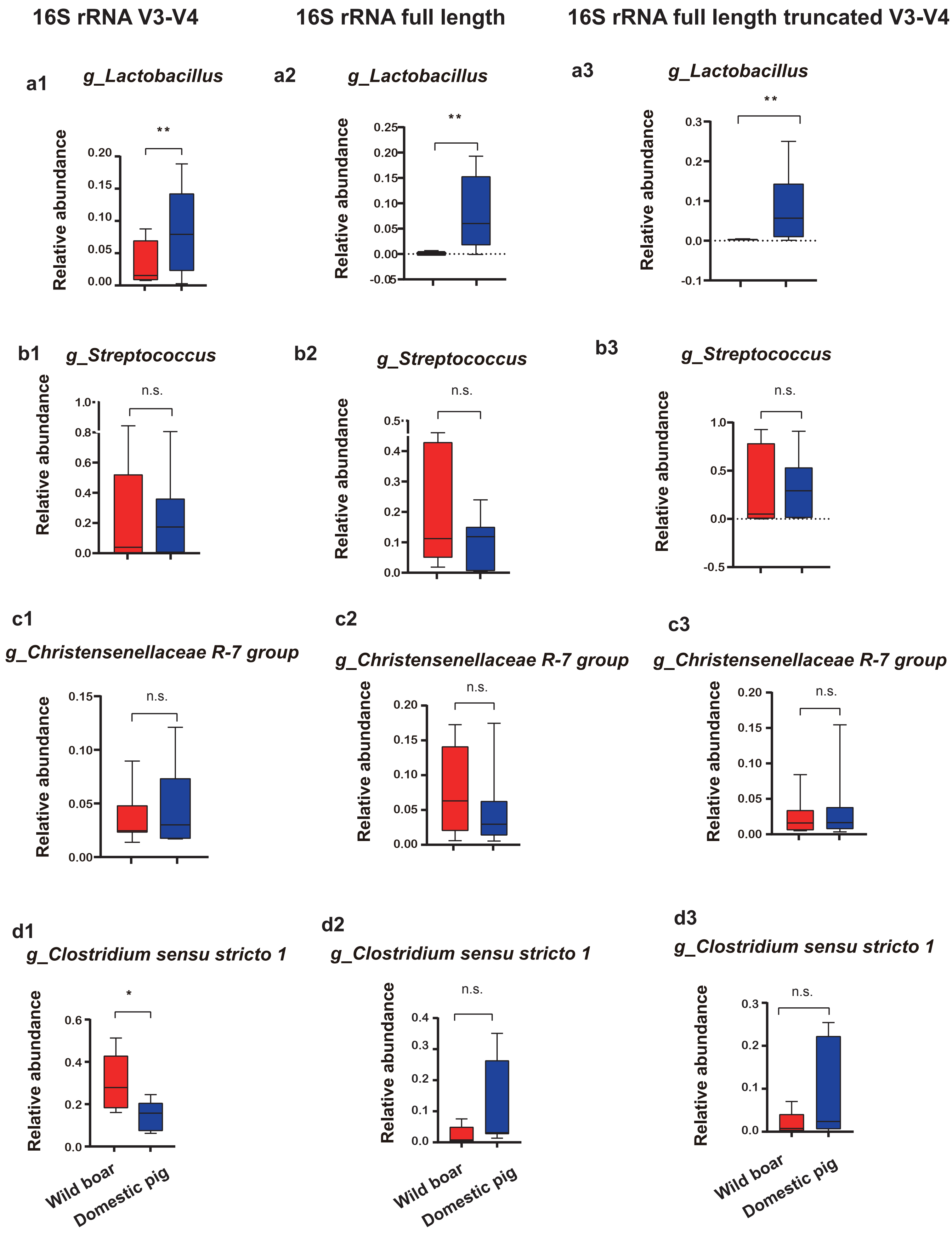

16S rRNA V3-V4
16S rRNA full length
16S rRNA full length truncated V3-V4
a1
g_Lactobacillus
a2
g_Lactobacillus
a3
g_Lactobacillus
b1
g_Streptococcus
b2
g_Streptococcus
b3
g_Streptococcus
c1
g_Christensenellaceae R-7 group
c2
g_Christensenellaceae R-7 group
c3
g_Christensenellaceae R-7 group
d1
g_Clostridium sensu stricto 1
d2
g_Clostridium sensu stricto 1
d3
g_Clostridium sensu stricto 1
Relative abundance
**
*
n.s.
Wild boar
Domestic pig
