# Supplementary figures and images for: Comparison of Changes in Gut Microbiota in Wild Boars and Domestic Pigs Using 16S rRNA Gene and Metagenomics Sequencing Technologies
Source: Animals (Basel). 2022 Sep 1;12(17):2270. doi: 10.3390/ani12172270 (PMC9454828; doi:10.3390/ani12172270)

**A**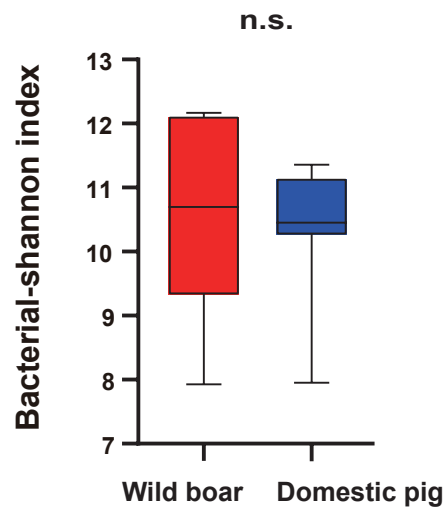**B**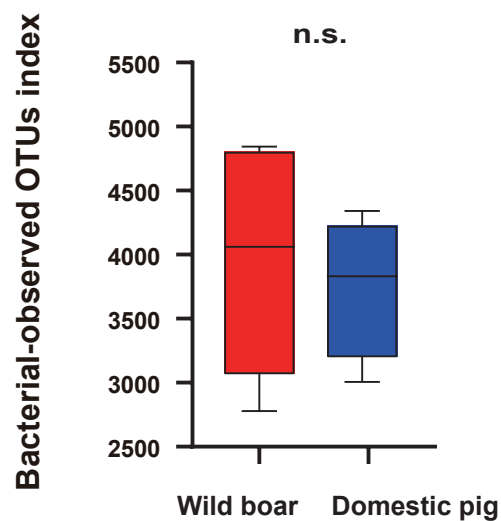**C**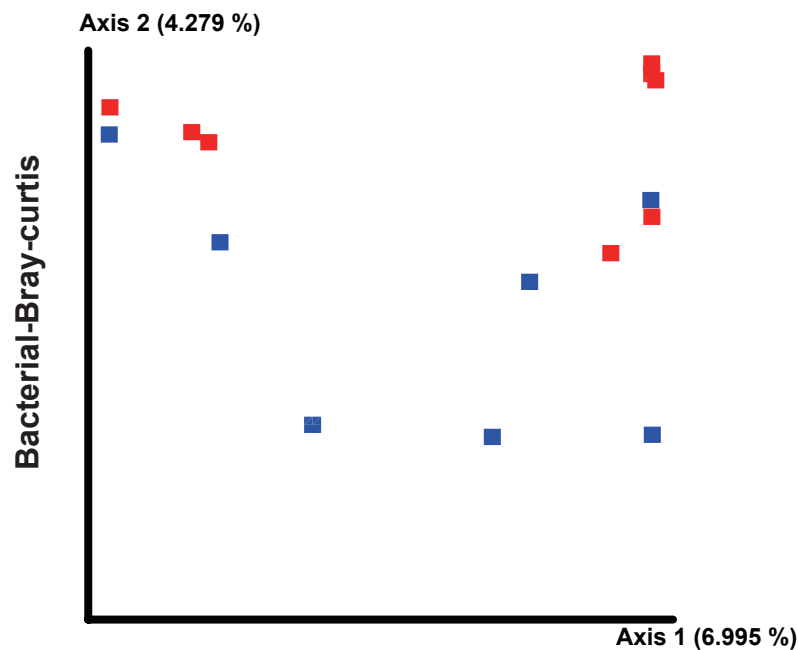**D**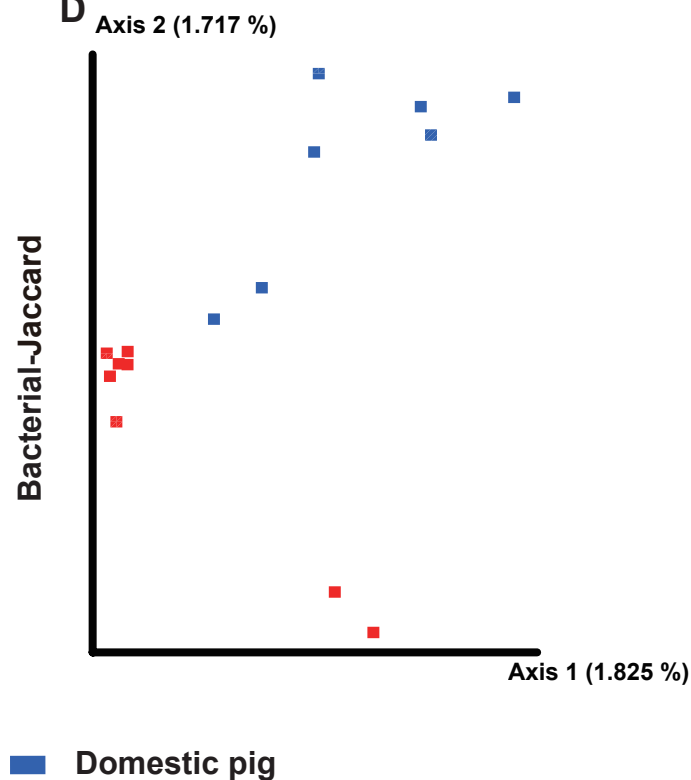

Supplement: Supplementary file 1 [file animals-12-02270-s001.zip › animals-1802100-Supplementary/Fig.S6.pdf]

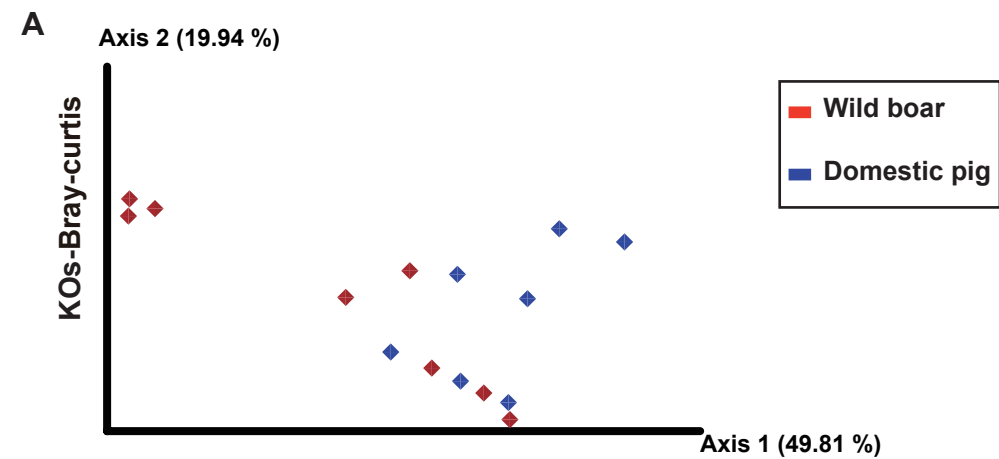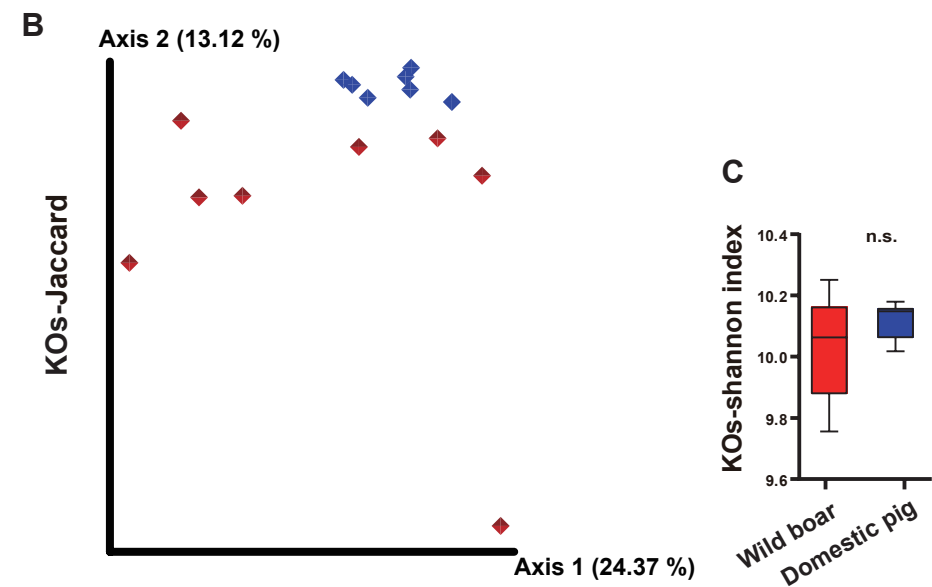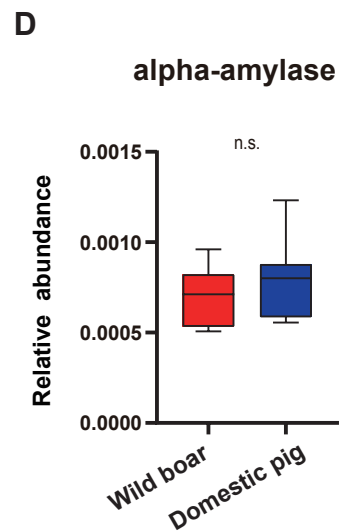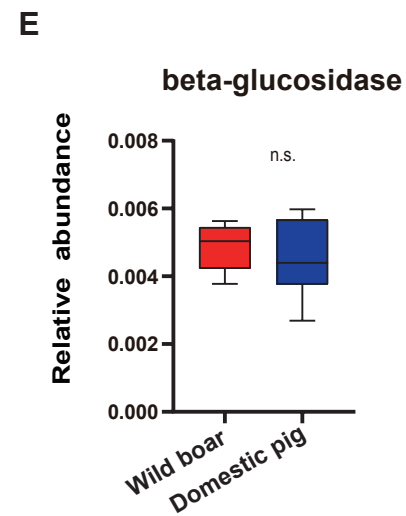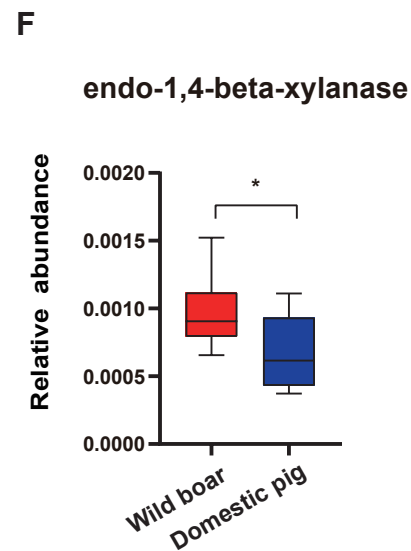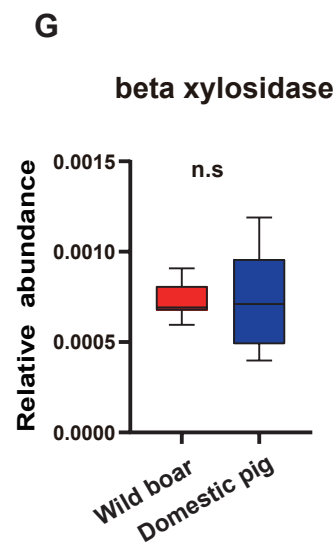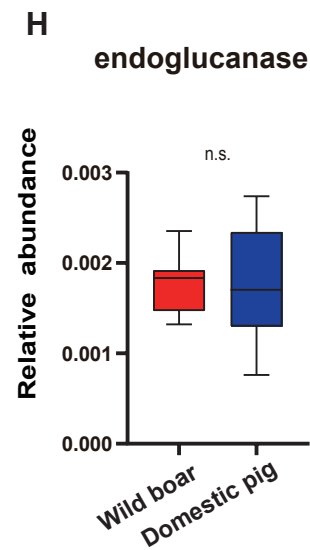

Supplement: Supplementary file 1 [file animals-12-02270-s001.zip › animals-1802100-Supplementary/Fig.S7.pdf]
